# Conception and prospective multicentric validation of a Robotic Surgery Training Curriculum (RoSTraC) for surgical residents: from simulation via laboratory training to integration into the operation room

**DOI:** 10.1007/s11701-023-01813-6

**Published:** 2024-01-27

**Authors:** Michael Thomaschewski, Markus Kist, Markus Zimmermann, Claudia Benecke, Jörg C. Kalff, Colin M. Krüger, Benno Mann, Andreas Türler, Tobias Keck, Richard Hummel

**Affiliations:** 1 Department of Surgery, University Medical Center Schleswig-Holstein, Campus Lübeck, Lübeck, Germany; 2 Department of General, Visceral, Thorax and Vascular Surgery, University Hospital Bonn, Bonn, Germany; 3 University Clinic Rüdersdorf, Brandenburg Medical School Theodor Fontane, Rüdersdorf, Germany; 4 Clinic for Visceral Surgery, Augusta-Kranken-Anstalten Bochum, Bochum, Germany; 5 Department of General and Visceral Surgery, Johanniter-Kliniken Bonn GmbH, Bonn, Germany

**Keywords:** Robotic surgery training, Training robotic surgery basic skills, Validation of ex vivo robotic surgery training, Transferability of robotic surgery training, Robotic training curriculum

## Abstract

There is a lack of training curricula and educational concepts for robotic-assisted surgery (RAS). It remains unclear how surgical residents can be trained in this new technology and how robotics can be integrated into surgical residency training. The conception of a training curriculum for RAS addressing surgical residents resulted in a three-step training curriculum including multimodal learning contents: basics and simulation training of RAS (*step 1*), laboratory training on the institutional robotic system (*step 2*) and structured on-patient training in the operating room (*step 3*). For all three steps, learning content and video tutorials are provided via cloud-based access to allow self-contained training of the trainees. A prospective multicentric validation study was conducted including seven surgical residents. Transferability of acquired skills to a RAS procedure were analyzed using the GEARS score. All participants successfully completed RoSTraC within 1 year. Transferability of acquired RAS skills could be demonstrated using a RAS gastroenterostomy on a synthetic biological organ model. GEARS scores concerning this procedure improved significantly after completion of RoSTraC (17.1 (±5.8) vs. 23.1 (±4.9), *p* < 0.001). In step 3 of RoSTraC, all participants performed a median of 12 (range 5–21) RAS procedures on the console in the operation room. RoSTraC provides a highly standardized and comprehensive training curriculum for RAS for surgical residents. We could demonstrate that participating surgical residents acquired fundamental and advanced RAS skills. Finally, we could confirm that all surgical residents were successfully and safely embedded into the local RAS team.

## Introduction

Robot-assisted surgery (RAS) is an innovative technology in the context of minimally invasive surgery (MIS). RAS is a completely new type of surgery that has the potential to combine the advantages of open surgery with those of laparoscopic surgery. With this new approach, previous limitations of laparoscopic surgery could be overcome, thereby enabling more patients to benefit from MIS. However, at the same time, in RAS there are several new challenges such as handling the interfaces of robotic systems, the altered or complete loss of haptics, and the new ways of communication in the surgical team. The surgeon must be able to master all these new features to take full advantage of robotics in MIS. In contrast, the uncontrolled or incorrect use of RAS entails risks and hazards leading to governmental and juristic limitations in the ability to use innovative technology as shown previously for laparoscopic surgery [1]. Therefore, various training curricula for laparoscopic surgery have been developed and implemented. Several studies on laparoscopic surgery training have shown that the skills learned on a simulator, on a box trainer or on synthetic organ models can be transferred to the operating room (transferability), resulting in a significant reduction in complications and in shorter operating times [2–6]. Although data on RAS in this regard are much more limited, initial studies also confirm the need for RAS training outside the operating room [7–11].

Based on historical experience and current reports on introduction of new technical systems into surgical practice, standardized training programs should be in place to ensure safe use and avoid hazards. Even more, as robotic surgery is increasingly used in MIS and might become a standard of treatment in many surgical fields, it is important to ensure broad training of the new technology, including surgical residents. In particular, to date, there are no training curricula focusing on the training of surgical residents in this new technology.

Here, we describe the conception of a structured and comprehensive RAS training program for surgical residents in visceral surgery: the Robotic Surgery Training Curriculum (RoSTraC).

RoSTraC specifically aims to provide surgical residents with multimodal learning of RAS, from simulation to laboratory training to integration into the robotic surgery team in the operating room. To demonstrate feasibility of the proposed curriculum and the transferability of acquired skills to RAS procedures, we conducted a prospective multicenter study with 7 surgical residents in 5 RAS centers nationwide.

## Materials and methods

### Conception of RoSTraC

Conception of RoSTraC included several aspects that are in our opinion relevant for structured and effective RAS training for surgical residents.

First, we aimed to provide RAS training on a ***self-contained basis*** allowing the trainees to work at their own pace and at the robotic system in their own institution without the need for extensive traveling, high costs of training courses or permanent supervision. Synthetical models are important in this context as usability of the clinical system was not hampered by the use of potentially infectious cadaveric or animal models. To implement training on a self-contained basis, we established an interactive cloud-based learning platform for RoSTraC. The cloud provides access to theoretical basics of RAS, instructions, and video tutorials of the RoSTraC exercises. At the same time, the trainees can upload their learning progress via the cloud for documentation and assessment of their RAS performance status. Even more, the cloud can be interactively filled with further content, e.g. video lectures, which are created by the trainees within the curriculum. Google Drive (Google LCC) and iCloud (Apple Inc.) were used for file hosting services. In addition, regular video meetings were established with the participants of RoSTraC to share each other’s learning progress, to present the video lectures on the theoretical basics and to analyze videos of RAS procedures step by step.

**Fig. 1:**
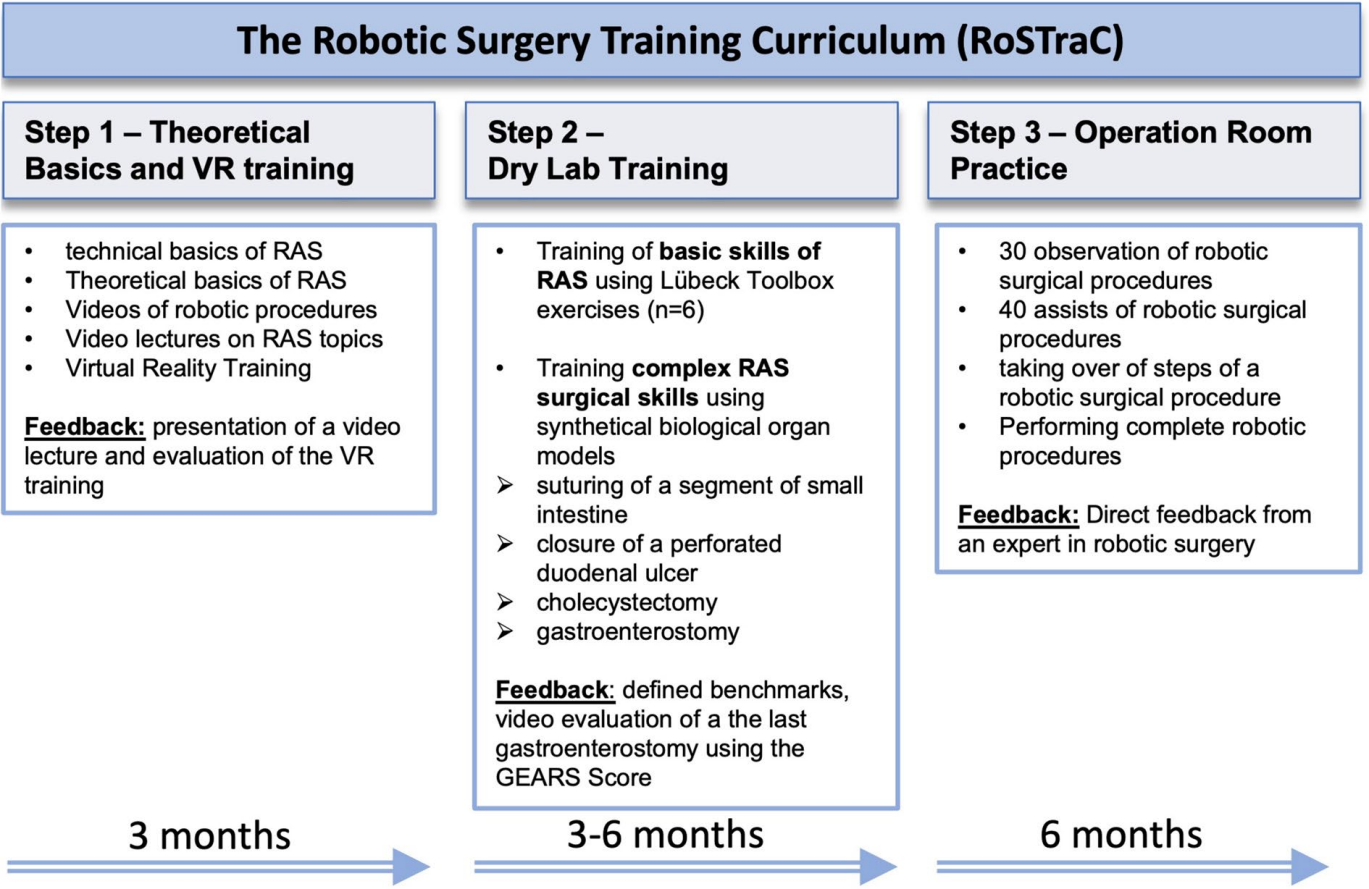
The structure of RoSTraC

Second, the curriculum should enable surgical residents to become part of the ***in-clinic robotic surgical team***. To ensure this in a safe way, the curriculum provides multimodal RAS learning contents including theoretical basics, RAS basic skills, complex RAS skills, and operation room training. To clearly structure the multimodal RAS training, RoSTraC was divided into three steps. The first step consists of theory and VR training. The second step includes an in vitro laboratory (lab) training on training boxes and synthetic biological organ models for the training of basic and complex RAS skills. In the last step, training takes place in the operating room including observation and assisting in robotic procedures and performing surgical steps on the *console*, all of which can be completed over a period of about 1 year (Fig. 1).

### Step 1—Theoretical basics and simulation

The first step of RoSTraC includes the theoretical basics about the following RAS topics: available robotic systems, the evidence of RAS and the economics of RAS. The theoretical basics of the RAS topics are provided on the one hand by selected literature and on the other hand by video lectures on the above RAS topics, which are created and presented by the trainees at the regular video meetings. In addition, these video lectures are made available to further trainees via upload into the cloud. In addition, step 1 of RoSTraC includes RAS video training that explains step-by-step RAS procedures in visceral surgery. Parallel to the acquisition of the theoretical basics of RAS, a technical introduction to the in-clinic robotic system is provided by the manufacturer of the robotic system. As transferability of the curriculum to any existing device was within the scope of the curriculum this part is mainly dependent on close cooperation with the manufacturer of the robotic system. Therefore, the trainees complete the VR training provided by the manufacturer of the robot system. Successful completion of the first step of RoSTraC requires a successful completion of VR training. Step 1 takes about 3 months to complete (Fig. 1).

### Step 2—Laboratory training

The second step of RoSTraC consists of a laboratory (lab) training using different training modalities such as training boxes and synthetic biological organ models at different levels of complexity and difficulty. All these exercises are carried out with the on-site robotic system in the operating room using the console, patient cart, video cart, a box trainer and training instruments.

**Fig. 2:**
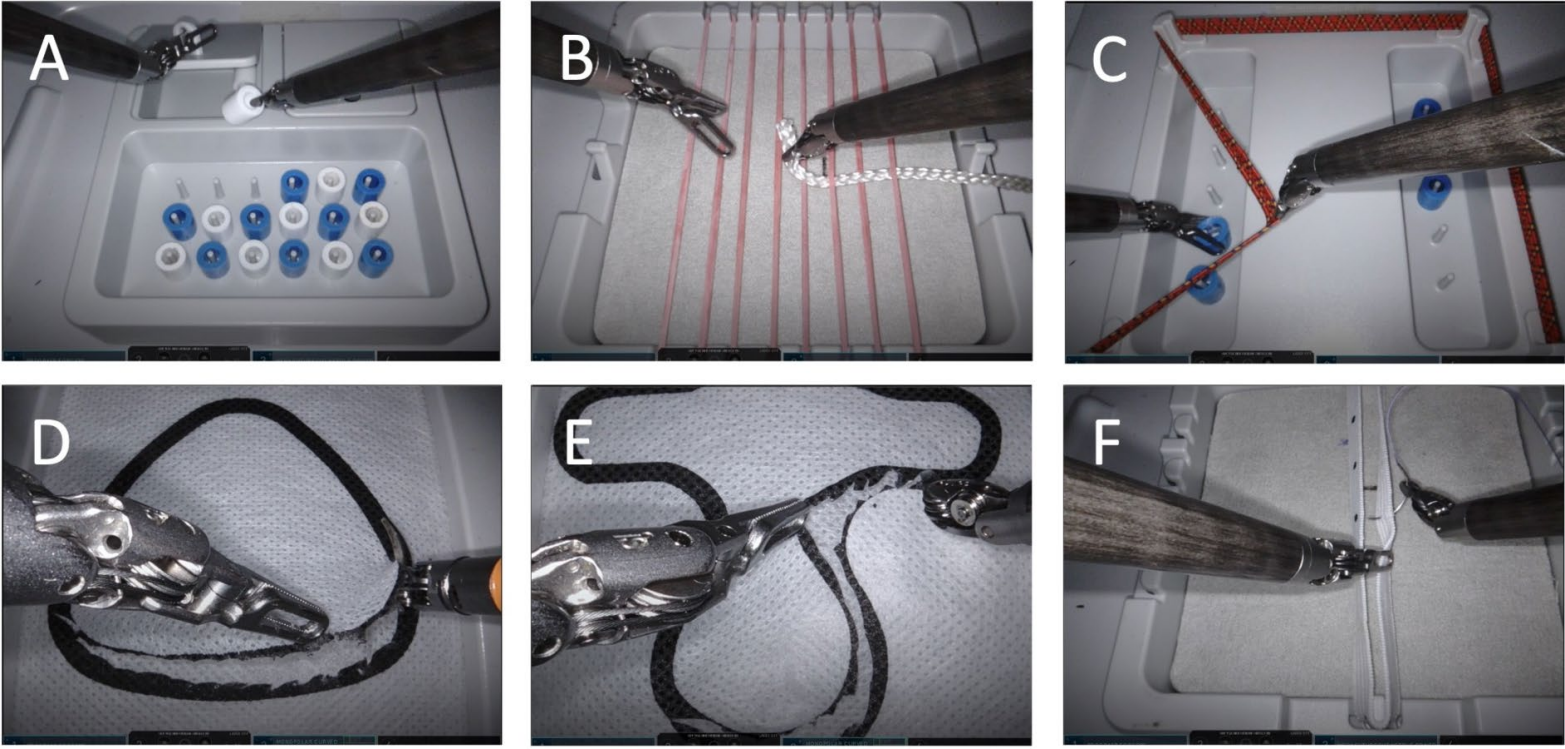
Lübeck Toolbox (LTB) exercises modified for RAS surgery. (**A**) Pack your luggage, (**B**) Weaving, (**C**) Nylon twist, (**D**) Triangular cut, (**E**) Hammer cut, (**F**) Suture

At first, RAS basic skills are learned which include bimanual coordination, handling the complete loss of haptics, usage of instruments with increased degrees of motion freedom, coordination of camera and general handling of the robotic system. For the acquisition of these RAS basic skills, we used the “Lübeck Toolbox” (LTB) exercises as previously described [6] (Fig. 2). Video tutorials have been created for each of the six LTB exercises, explaining how to perform the exercise correctly. For LTB exercises A, B, C and F, a robotic grasping instrument and a robotic needle holder were used. A robotic grasping instrument and a robotic scissors were used for exercises D and E. The exercises had to be repeated until the objectives of the exercises (expert level) were reached in at least 2 repetitions.

**Fig. 3:**
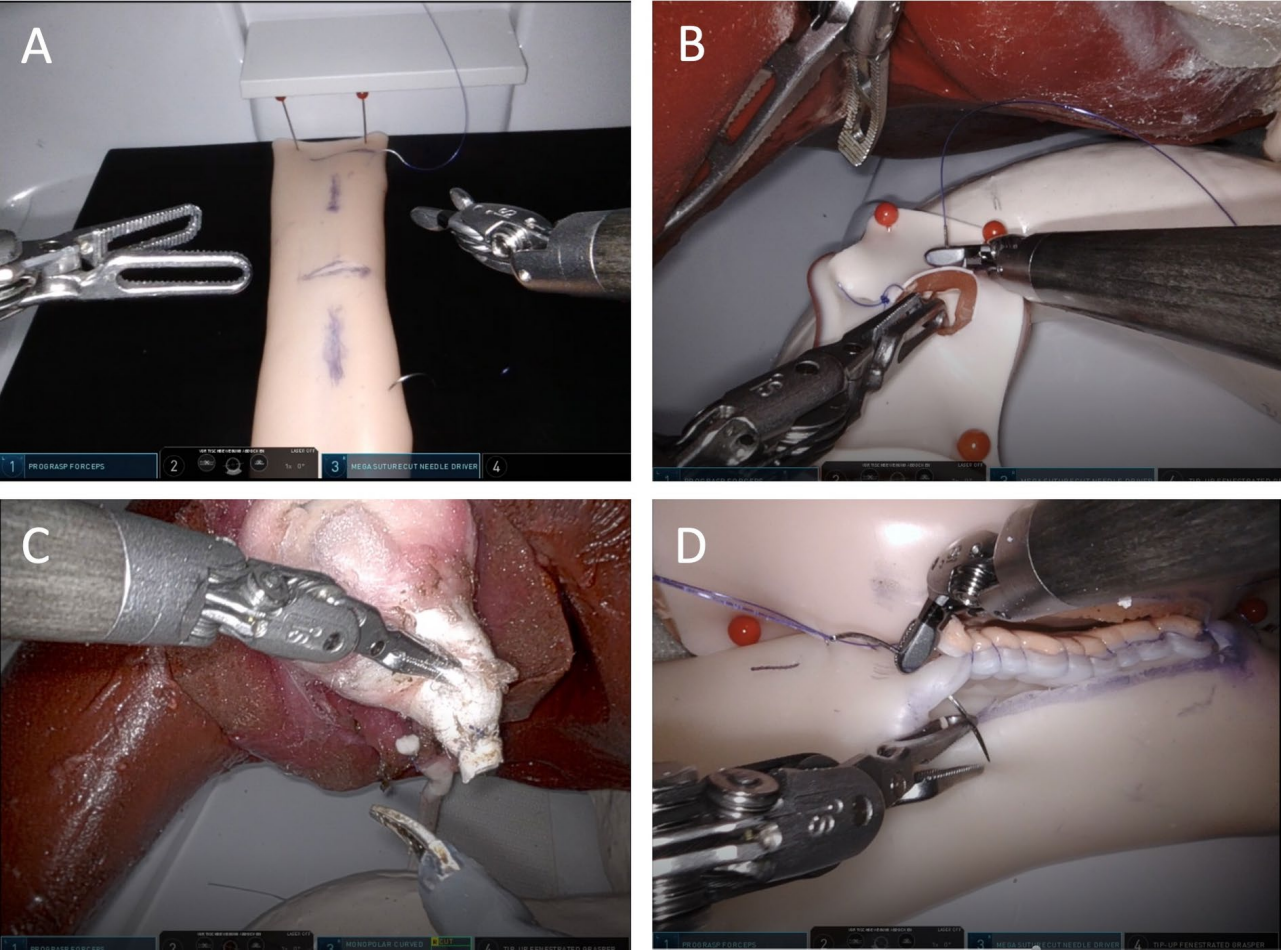
Robotic procedures on synthetic biological organ models (No. 1–4.) (**A**) No. 1: suturing of a segment of small intestine, (**B**) No. 2: closure of a perforated duodenal ulcer, (**C**) No. 3: cholecystectomy, (**D**) No. 4: lateral-lateral gastroenterostomy

After the acquisition of RAS basic skills, four exercises with increasing levels of complexity are performed on synthetic biological organ models. The synthetic biological organ models consist of a synthetic human torso and synthetic organs of the upper abdomen (HumanX GmbH, Wildau, Germany) (Fig. 3). In particular, the exercises on synthetic organ models mimic realistic robotic operations, namely suturing an enterotomy in the small intestine, a cholecystectomy, a closure of a perforated duodenal ulcer and a latero-lateral gastroenterostomy. These synthetic organ models can be used without hygienic limitations in every hospital. In the following, the individual exercises on the synthetic organ model are explained in detail.

In the first exercise (No. 1), a 5 cm enterotomy in the small intestine must be closed with a continuous suture in both the horizontal and vertical directions (PDS II 4–0 (Ethicon Inc.) shortened to a length of 8 cm) with anchoring knots (Fig. 3A). This procedure is successfully completed when the target time of 6:15 min is reached (expert level, see benchmark study) and the exercise has been repeated at least 20 times. For this procedure, one robotic grasping instrument and one robotic needle holder are used.

The subsequent exercises (exercises No. 2–4) train not only RAS skills of increasing complexity including entire RAS procedures, but also the efficient use of the additional 4th robotic arm for exposure the surgical field. Two robotic grasping instruments, a robotic needle holder and a monopolar scissors are used for these exercises.

In exercise No. 2 (Fig. 3B) on the synthetical biological organ model, a perforated duodenal ulcer is closed with three single sutures each using a PDS II 4-0 (Ethicon Inc.) suture shortened to 8 cm while lifting the liver that covers the duodenum using the fourth robotic arm.

Next, a cholecystectomy is practiced (exercise No 3; Fig. 3C). Using the additional fourth robotic arm, the gallbladder is exposed. Analogous to the principles of MIS cholecystectomy, a dissection of Calot’s triangle is performed. The cystic duct is cut between two ligations (Vicryl 4-0, shortened to 8 cm (Ethicon Inc.)). Finally, the gallbladder is dissected from the liver with the monopolar scissors.

Finally, a lateral-lateral gastroenterostomy is performed as the most complex exercise (exercise No. 4; Fig. 3D). The stomach is first exposed by lifting the liver with the additional fourth robotic arm. Then, a loop of small intestine is sutured to the large gastric curvature with a self-anchoring suture (V-Loc™, Covidien/Medtronic) over a distance of about 4–6 cm. This is followed by a gastrostomy and enterotomy over a distance of 4 cm using the monopolar scissors. Lateral-lateral gastroenterostomy is then performed with a double-armed PDS II 4-0 suture (Ethicon Inc.). First, the posterior wall is sutured continuously, followed by the anterior wall. Both ends of the suture are finally knotted. The closure of a perforated duodenal ulcer, the cholecystectomy, and the gastroenterostomy are repeated 5 times each. For standardization and comparison, all exercises and surgical procedures must be performed exactly as instructed in the video tutorials. To test the acquired RAS skills and to provide feedback to the trainee on her/his RAS skills, the last robotic gastroenterostomy will be recorded, uploaded to the cloud and evaluated by an expert in robotic surgery using the validated multidimensional *Global Evaluative Assessment of Robotic Skills* (GEARS) score [12]. Step 2 takes about 3–6 months to complete (Fig. 1).

The choice of content for the exercises in step 2 was based on the following considerations: First, we integrate toolbox exercises from the LTB in step 2 to teach the basic skills of MIS, such as how to deal with altered or lacking haptics. The contents of the LTB have already been validated in previous studies [6]. Next, we integrated exercises on synthetic organ models in step 2 to train a RAS procedure ex vivo in a safe environment before step 3 of the RoSTraC, where RAS procedures are performed in the operating room on the patient. The RAS procedures on the organ models teach the skills required for step 3, such as the dissection of tissue in the correct layers, the effective use of the additional robotic arm in exposing the surgical situs and the suturing of anastomoses.

### Step 3—Operation room practice

In the final step, training takes place in the operating room during RAS procedures on patients. First, this step includes at least 30 observations and at least 40 assists in RAS procedures. This is followed by taking-over of individual minor steps of a RAS procedure (such as for example control of camera while changing focus, exposure of situs with four arms, minor cutting or suturing steps, mobilization of for example the ascending/descending colon or similar) at the console under the supervision of an experienced robotic surgeon. The type of procedures is based on the specialty of the trainee’s surgical clinic. In the further course of the training, the trainee has to perform a minimum of 5 major sub steps of complex operations, or simple small operations. Examples are mobilization of the colon (including the right or left colonic flexure), hiatoplasty, gastric wedge resection, sigmoid resection or similar. This part of training aims at a more extensive exposure to RAS under supervision. This step finally leads to the trainees becoming an integral part of the robotics team in their own clinic. This step takes about 6 months to complete (Fig. 1). It should be noted that in RoSTraC the observations and assists in RAS procedures do not necessarily have to take place after the successful completion of step 2, but can take place parallel to step 2. However, taking-over individual steps of an RAS procedure in the operation room should only be performed after successfully passing step 2.

### Benchmark study to determine the expert level

To determine an expert level for the LTB exercises and the robotic procedure of suturing of a segment of small intestine, four experienced robotic surgeons from two surgical centers were recruited. These surgeons, each, had performed more than 50 RAS procedures. For standardization, all experts received access to the RoSTraC cloud and watched the video tutorials explaining the exercises. Then, all exercises were repeated three times subsequently. Each repetition was performed respecting the pre-defined precision. For the definition of the expert levels or the respective exercises, the mean values of the execution times of all experts (in seconds) were calculated.

### Validation study

A prospective multicenter study was conducted to validate RoSTraC by determining the learning curves and demonstrating the transferability of the acquired skills to a RAS procedure. The prospective trial was approved by the ethics committee of the University of Lübeck (#2022-568). Study participants were surgical residents in the last years of their surgical residency and surgeons who have just completed their residency in general or visceral surgery. Seven study participants from five different surgical clinics in Germany were recruited for the validation study. All study participants signed informed consent. The study was conducted on-site at the surgical clinic where the study participants were undergoing their surgical residency for general and/or visceral surgery. All participating surgical clinics had a *DaVinci Surgical Si* or a *DaVinci Surgical Xi* system (Intuitive Surgical Inc.) and provided equipment to perform the RoSTraC. LTB training boxes and synthetic biological organ models for step 2 of the curriculum were provided by the study center (*University Medical Center Schleswig–Holstein Campus Lübeck, Department of Surgery*). The study participants completed RoSTraC as described above. The VR training in step 1 of RoSTraC was performed using the *DaVinci Skills Simulator* (Intuitive Surgical Inc.) as defined by Intuitive Surgical Inc. including a total training time of 40 h. As baseline of RAS performance, all study participants performed a robotic gastroenterostomy on the synthetic biological organ model as described above after completing the first step of RoSTraC (pre-test/GE I). Following the pre-test, the study participants completed the second step of RoSTraC as described above. For the evaluation of the learning curves, the study participants documented the repetition times of all exercises in step 2 and uploaded them to the RoSTraC cloud. GE I (pre-test) and the last robotic gastroenterostomy at the completion of step 2 of RoSTraC (GE II) were digitally recorded and the videos were uploaded into the RoSTraC cloud for assessment by the study center. Due to technical limitations on unsuccessful video recording, video evaluation was only possible for 5 out of 7 participants. Video assessment of GE I and GE II was performed by three RAS experts using the multidimensional GEARS score [12]. The determination of the GEARS scores by the three RAS experts was performed blindly and independent from each other. Changes from GE I to GE II were analyzed.

In step 3 of RoSTraC, the study participants documented the number of assists of RAS procedures on the *patient cart* and the number and type of performed steps of RAS procedures on the *console* and uploaded data to the RoSTraC cloud.

Study parameters included gender, age, level of education of study participant, execution times of the RoSTraC exercises, GEARS Score of GE I and GE II (including the five domains depth perception, bimanual dexterity, efficiency, tissue handling and autonomy) and number and type of surgical procedures and/or steps of surgical procedures performed on the *console* in the operating room on patients.

### Evaluation study

The evaluation of the RoSTraC curriculum was performed using a questionnaire answered by the study participants of the validation study (evaluation study). SurveyMonkey® was used to conduct the evaluation study and to analyze the responses. The questions were answered anonymously by the study participants. The questions included 2 topics: (1) In the first topic, study participants were asked how many repetitions of the *DaVinci Skills Simulator*® exercises (Intuitive Surgical Inc) were necessary to achieve the targets and after how many hours all targets had been achieved. (2) In the second topic, RoSTraC was evaluated in terms of relevance to clinical practice, structure, and learning content. Finally, the curriculum was assessed, awarding an overall grade. In addition, the participants were asked whether they completed the training curriculum primarily during working hours or during their free time.

### Statistics

Data processing and statistical analysis were performed using Excel (*Microsoft Corporation*). Ordinal and nominal variables were expressed as absolute numbers and percentage. Bar charts were used to visualize results. Learning curves were calculated with Excel (*Microsoft Corporation*). For comparison of GE I and GE II according to the GEARS Score, the paired sample t-test was used. The changes from GE I to GE II were indicated by mean values and standard deviations. For all statistical analyses, a *p* value of *p* ≤ 0.050 was considered significant.

## Results

### Benchmark study to determine the expert level

The results for determination of the expert level are shown in Table 1 (benchmark study).

**Table 1:** Definition of an expert level for RoSTraC exercises

RoSTraC exercise	Mean repetition time of expert’s performance [sec. ± SD]
No. 1 “pack your luggage”	82 (±7)
No. 2 “weaving”	48 (±15.4)
No. 3 “nylon twist”	117 (±26.9)
No. 4 “triangular cut”	69 (±16.1)
No. 5 “hammer cut”	120 (±37.7)
No. 6 “suture”	52 (±11.6)
No. 7 “suturing a segment of small intestine”	375 (±10.5)

### Validation study

Seven study participants from five RAS centers in Germany participated in the validation study. The mean age of study participants was 34 years (31–41 years), with 64.3% male and 35.7% female. 69.2% of study participants have completed their surgery residency training in visceral surgery within the last year in average (range since completion of training: 0–70 months), and 30.8% were surgical residents in their final years. All study participants were right-handed. 30.7% of study participants play a musical instrument and 15.4% play video games regularly (once a week).

All study participants met the objectives of the VR training on the *DaVinci Skills Simulator*^®^. The study participants needed in median 3 repetitions (range 1–9) to achieve the targets of the *DaVinci Skills Simulator*^®^ exercises (DaVinci Intuitive Surgical Inc). In average, after 8 h of VR training, all targets of the *DaVinci Skills Simulator*^®^ were successfully achieved.

For the LTB exercises, study participants required a median of 3 (range 2–5) repetitions for exercise A “pack your luggage”, 4 (range 2–6) repetitions for exercise B “weaving”, 3 (range 2–7) repetitions for exercise No. C “nylon twist” and 4 (range 3–5) repetitions for exercise D “triangle cut” to achieve the defined target times (expert level). More repetitions were required for exercise E “hammer cut” with a median of 6 repetitions (range 4–10). For the last LTB exercise F “suturing”, a median of 9 (range 7–12) repetition were required to achieve the defined target times (expert level).

**Fig. 4:**
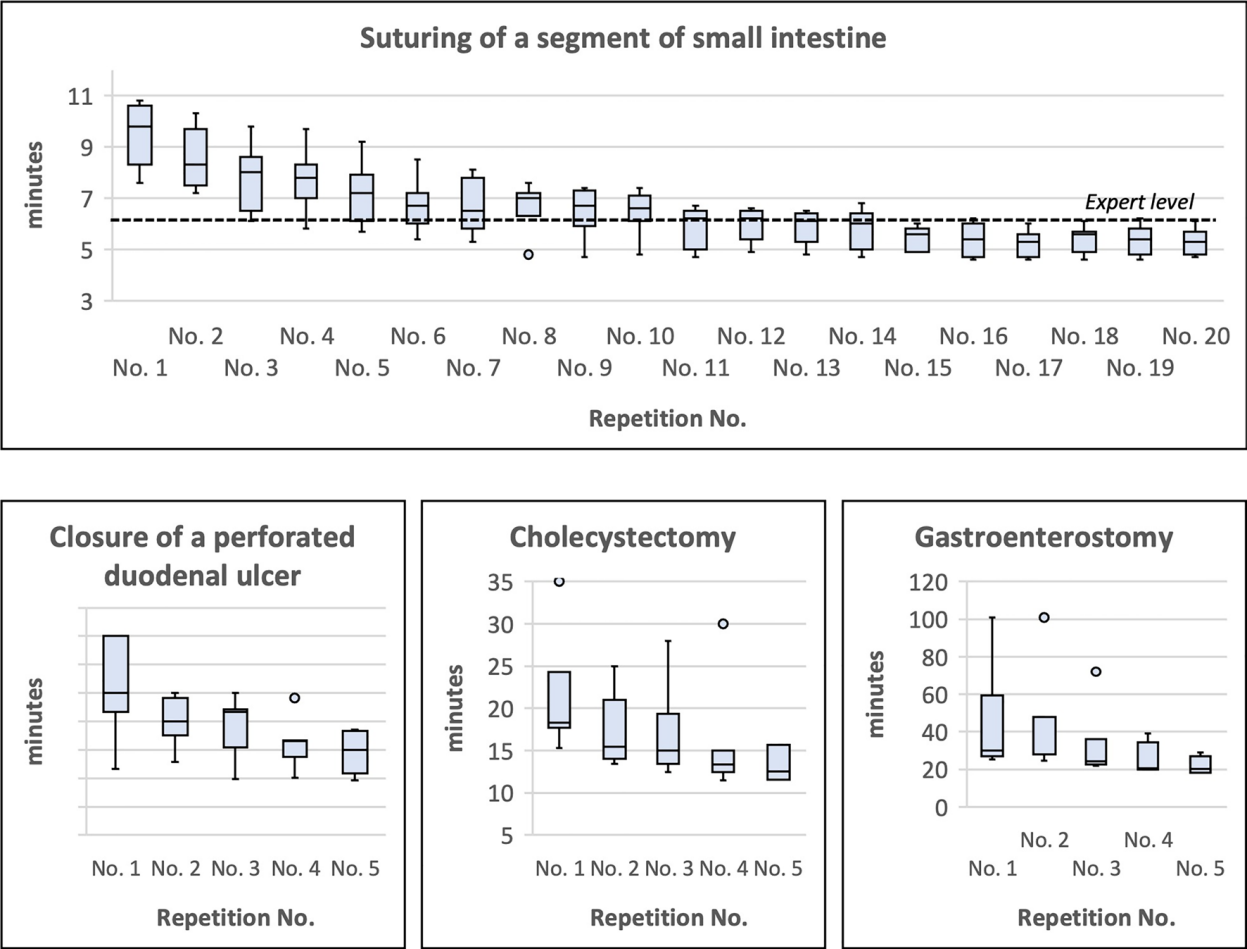
Learning curves of RAS procedures using synthetical biological organ models

The learning curves of the RAS procedures on the synthetic biological organ models are shown in Fig. 4. For the RAS procedure “suturing of a segment of small intestine”, the median number of repetitions to reach the objectives (expert level) was 11 (range 3–15). At the beginning of the learning curve, there is a steady improvement in execution times until a plateau phase was reached after approximately 11 repetitions. The value of the benchmark study (expert level: 375 s) was located at the beginning of the plateau phase of the learning curves of study participants. The learning curves of the other 3 robotic procedures on the synthetic biological organ models, which were repeated 5 times each, showed a steady improvement in the execution times. However, a plateau phase was not reached within the 5 repetitions in the learning curve (Fig. 4).

**Fig. 5:**
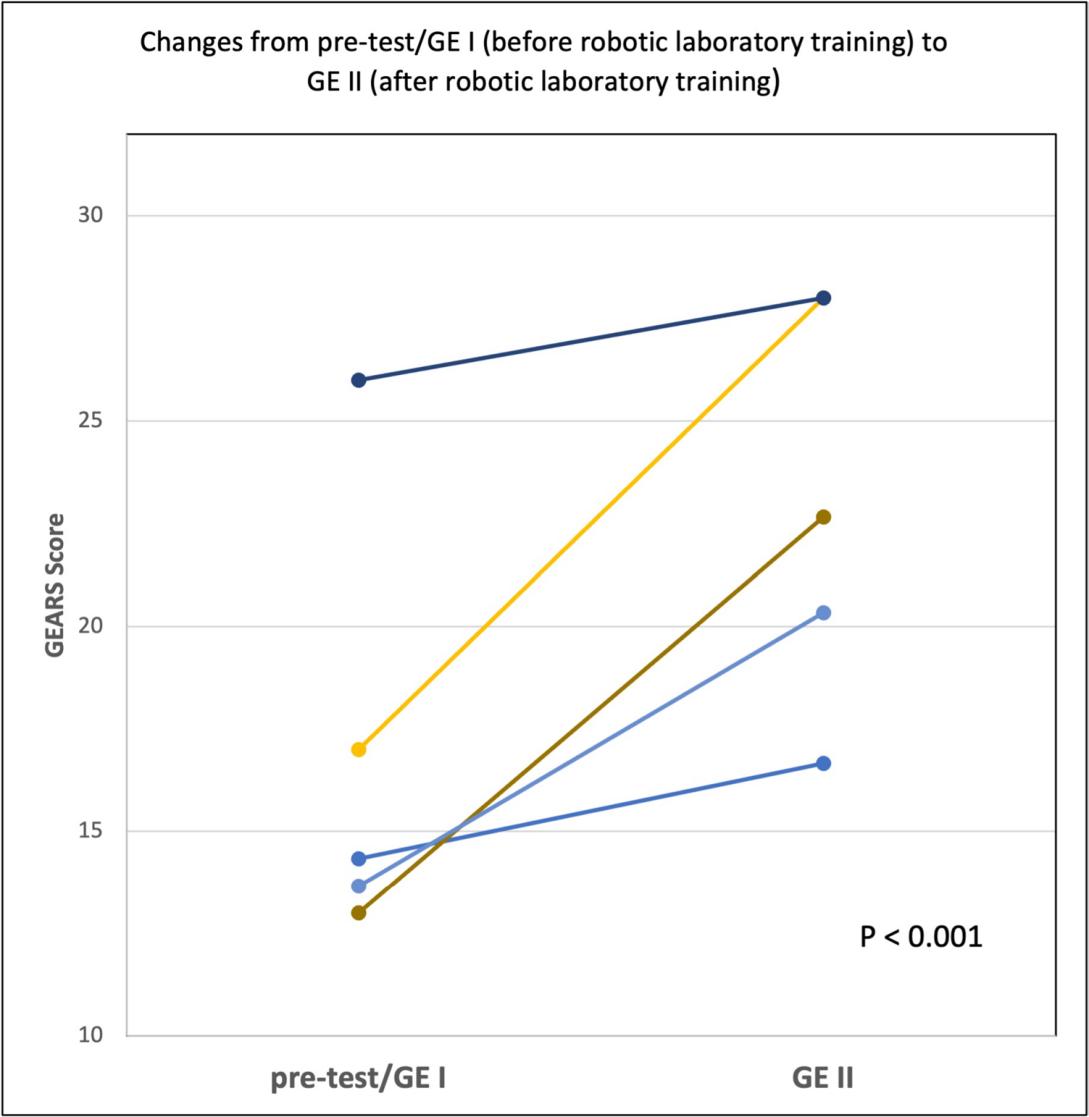
Changes and/or improvements from pre-test (GE I) before robotic laboratory training (step 2 of RoSTraC) to GE II after robotic laboratory training (step 2 of RoSTraC) for 5 out of 7 participants. Blinded evaluation of the robotic gastroenterostomies before (GE I) and after (GE II) step 2 showed a significant improvement of RAS performance from a mean GEARS score of 17.1 (±5.8) points to 23.1 (±4.9) points (*p* < 0.001)

Blinded evaluation of the robotic gastroenterostomy before (pre-test/GE I) and after (GE II) step 2 of RoSTraC revealed a mean GEARS score of 17.1 (±5.8) points and 23.1 (±4.9) points, respectively (Fig. 5). This improvement in GEARS score was statistically significant with *p* < 0.001. In addition, surgical participants showed a significant improvement in the RAS performance in all domains of the GEARS score including depth perception, bimanual dexterity, efficiency, force sensitivity, robotic control, and autonomy.

With regard to Step 3, “operation room practice” all study participants performed the required number of 30 observations and 40 assists in RAS procedures in the operating room within six months after completion of step 2 of RoSTraC. Furthermore, all study participants performed RAS procedures or major steps of RAS procedures at the console on patients under supervision of an expert in RAS. During the six months following the completion of step 2, study participants performed a median of 12 (range 5–21) RAS procedures on the console including both general surgical and oncological RAS procedures (Table 2).

**Table 2:** Number of robotic procedures performed by a surgical resident in the operating room at the robotic console *(HPB: Hepato-pancreato-biliary GI: gastrointestinal)*

Surgical specialty	Median number of robot-assisted procedures performed by a surgical resident
General surgery	4 (range 2–6)
Colorectal surgery	5 (range 2–8)
Upper GI and HPB surgery	2 (range 0–11)
Total	12 (range 5–21)

### Evaluation study

**Fig. 6:**
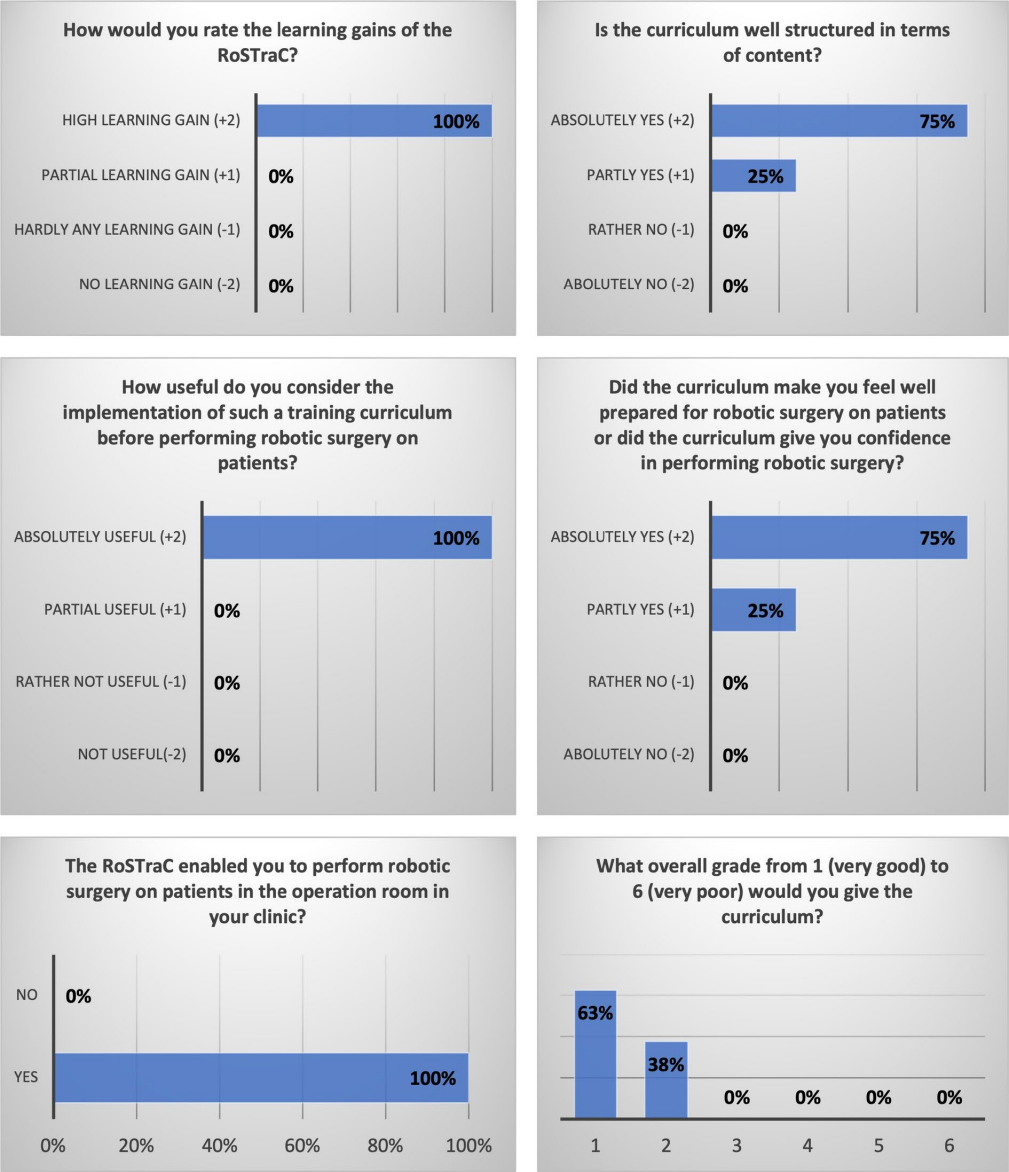
Results of the evaluation study of RoSTraC: a survey answered by the study participant after completion of RoSTraC

After successful completion of RoSTraC after one year, study participants evaluated the RoSTraC via an anonymous survey. The results of the questionnaire in terms of relevance to clinical practice, structure, and learning content are shown in Fig. 6. In the evaluation study, 25% of the study participants reported that they conducted the RoSTraC training both in their free time and during working hours. Most of the study participants (75%) reported that they conducted the training curriculum mainly in their free time.

## Discussion

We describe the first conception and prospective multicentric validation of a multimodal surgical training curriculum specifically designed for surgical residents. This curriculum teaches theoretical and practical competency in robotic surgery in general and visceral surgery. The Robotic Surgery Training Curriculum (RoSTraC) meets all the initially intended criteria as defined during conception:

First, we intended to design a RAS training curriculum for surgical residents that aims to integrate them into the in-clinic robotic surgical team and enable them to assist and perform RAS procedures in the operating room. Through its comprehensive and multimodal training structure, surgical residents are prepared for RAS operating room practice in an excellent way. In the validation study of RoSTraC, all study participants were able to perform steps of a robotic surgical procedure in the operation room on the console or even entirely robotic procedures (under supervision) including general surgery procedures as well as visceral oncological resections. In a national survey in the United States, 96% of surgical residents reported having access to a robotic system in their clinic, but only 18% of them had ever had experience with the robot console [13]. The results of the validation study showed that through the RoSTraC, surgical residents can successfully and safely be integrated into a RAS team. Thus, the RoSTraC enables and also ensures, a structured training of surgical residents at the console in the operation room. RoSTraC could herein also contribute to introduce more surgical residents to robotic surgery at an early stage of their training. As the use of robotic systems in general and visceral surgery continues to increase, a great need for training curricula in robotic surgery is existing. Even more, training curricula for robotic surgery could become an integral part of the surgical residency training. Finally, the RoSTraC provides a suitable method for implementing RAS into surgical residency training.

The *DaVinci Technology Training Pathway*, *Fundamentals of Robotic Surgery, The Society of American Gastrointestinal and Endoscopic Surgeon Robotic Masters Series*, *Fundamental Skills of Robot-Assisted Surgery training program*, and the *Robotics Training Network curriculum* are other published training curricula of robotic surgery [9]. However, their contents are inconsistent [9]. And these curricula are designed primarily for learning the basic principles of robotic surgery and do not include comprehensive training concepts in robotics enabling surgical residents to perform RAS procedures in the operation room at the console. On the other hand, there are structured RAS training curricula addressed to surgeons in advanced training in specific surgical fields. For example, Knab et al. developed a training curriculum for complex oncologic procedures in visceral surgery such as RAS pancreatoduodenectomy [14]. However, such training programs address experienced oncological surgeons and not surgical residents. Moreover, these training programs are often designed as part of a fellowship at a specialized clinic and not at the trainee’s own clinic.

Secondly, with the conception of RoSTraC we intended to provide training on a self-contained basis at the robotic system in the respective surgical clinic. Due to the limited time and personnel resources in the daily clinical routine, the time of a trainer for surgical education is often limited, which can have an unfavorable influence on the learning progress of the surgical residents. In addition, inadequate training leads to frustration of both the trainee and the trainer. For this reason, we aimed to design a training curriculum that enables surgical residents a largely independent, flexible and self-directed training in RAS. This is achieved in the RoSTraC primarily through the cloud-based accessibility of RAS learning and exercise contents (RoSTraC cloud). As an important element, the RoSTraC cloud includes video tutorials that teach the theoretical and practical basics of RAS to the trainee. Indeed, previous studies showed that video tutorials can partially replace direct in-person instruction and feedback [15–17]. Furthermore, the RoSTraC is designed to provide surgical training in the trainee’s own clinic using the in-house robotic system. Accordingly, there is no need for traveling to specialized external training centers. Even more, the RoSTraC is designed to be transferable to other robotic systems, which significantly increases the scope of the curriculum, especially as new robotic systems will be approved and used in visceral surgery in the near future. However, the independent training structure of RoSTraC using the in-house robot system may have the disadvantage that the training mainly takes place during free time instead of during working hours. Accordingly, the evaluation study showed that the training of the study participants mainly took place in their free time and not during working hours. There are two possible reasons for this to discuss: (1) Due to the limited resources of surgical residents in their daily clinical routine, the curriculum can only be completed in their free time. (2) Using the in-house robot system, training usually has to take place outside regular working hours, since within regular working hours the robot is used for routine operations. To solve this, suitable working time models are needed that enable training within working hours.

Moreover, RoSTraC meets several generally accepted requirements for training curricula [2–6, 18–20]: (1) the training curricula are clearly structured, (2) include clear objectives; (3) the learning content is validated and can be transferred to surgical procedures (validation and transferability); (4) the training curricula include feedback mechanisms and (5) are evaluated. RoSTraC meets all these requirements, providing a highly effective training program for learning RAS skills in general and visceral surgery:

*With regard to “clear structured training”*, in RoSTraC, RAS skill learning follows a 3-step process, with clear objectives at each learning step. The learning content of the exercises in the training lab builds on each other, starting with simple exercises that train bimanual handling to complex robotic surgical procedures that require effective handling of all robotic arms and devices. RoSTraC is completed over multiple training sessions over the course of one year. According to current studies on surgical training, a clearly structured training curriculum that is distributed over several training sessions is required not only to ensure effective learning progression, transferability, and adherence of what is learned, but also to keep the trainee’s motivation high [21]. In contrast, hands-on courses in which surgical skills are taught compactly in 1–2 days were inferior to training curricula distributed over several practice sessions [21].

With regard to the second requirement “*clear objectives*”, RoSTraC include exercises with clear objectives that were defined by a benchmark study with four experts in robotic surgery. Thus, by achieving these benchmarks, the trainee reaches an expert level. In laparoscopic surgery training, goal-oriented proficiency-based training has been shown to increase trainee motivation, including adherence to the curriculum, resulting in better performance compared with training without defined goals [22, 23].

With regard to the third requirement of “*validation and transferability*”, we could show in a multicentric validation study that the learning contents of RoSTraC exercises are valid and lead to a significant improvement in RAS performance (validation). In addition, we were able to show that the acquired RAS skills are transferable to a complex robotic procedure of a robotic gastroenterostomy (transferability): After performing the RoSTraC exercises and reaching the benchmarks, surgical trainees showed a significant improvement in the RAS performance of a robotic gastroenterostomy in all domains of the GEARS score (including depth perception, bimanual dexterity, efficiency, force sensitivity, robotic control and autonomy).

To address the fourth requirement “*feedback mechanisms*”, we defined benchmarks for RAS basic skills exercises and included video assessments of RAS exercises performed in the lab using the multidimensional GEARS Score*.* This provides trainees with feedback if they have already achieved the learning objective and in which domain or task they can further improve. Studies have shown that feedback mechanisms can make learning more effective [18–20]. Ideally, trainees should receive direct in-person instruction and feedback from the trainer when performing the surgical exercises. However, due to the limited personal resources in clinical practice, this is hardly feasible. Instead of direct feedback, alternative feedback mechanisms using defined benchmarks and video assessments have been implemented in RoSTraC.

Fifth, training curricula require an “*evaluation*” by the trainees to assess learning contents and learning progress from the trainees’ point of view*.* RoSTraC was evaluated by the study participants of the validation study through a survey (evaluation study). Here, the RoSTraC received top ratings in terms of structure, learning content, and relevance to surgical education. These results of the evaluation study further confirm the high quality and efficiency of RoSTraC also from the trainees’ point of view.

The following limitations need consideration for interpretation of the current study: the prospective multicenter validation study was performed only with the *DaVinci Xi* and *Si* systems. In principle, the RoSTraC is designed to be transferable to any other robotic system. However, whether the learning content of the RoSTraC is valid on other robotic systems and transferable to surgical procedures on other robotic systems remains unclear and needs to be investigated in additional studies where new benchmarks have to be established for each individual system. In the validation study, the number of study participants is comparatively small, and no statistical case number calculation was performed. Furthermore, we did not specifically investigate the impact of laparoscopic skills on the effect of training. However, based on their advanced surgical training (final year of training or finished specialty training), all participants presented with relevant laparoscopic skills. The robotic procedures on the synthetic biological organ model (closure of a perforated duodenal ulcer, cholecystectomy and gastroenterostomy) were each performed only 5 times based on the currently high costs of models. In the validation study, a plateau phase in the learning curve was not reached in any of these exercises during the 5 repetitions, so that more repetitions should be discussed in a cost benefit comparison. The difference in GEARS scores before and after step 2 of RoSTraC strongly suggests a transferability of the training effect to an almost realistic RAS procedure. Certainly, a limitation of our study is that we did not include a control group. It is therefore possible that the gain in GEARS values was merely the effect of one repetition of the gastroenterostomy performed after completion of all tasks. However, the study design of comparing the operative performance of a surgical procedure before and after the training curriculum without a control group has been also used in other studies to validate the learning content and transferability of a training curriculum [24]. Nevertheless, further studies are needed to prove the transferability especially to a real surgical procedure in the operating room [25].

### Conclusion

With this current manuscript we describe the conception and validation of the highly standardized and strictly defined proficiency goal-directed RoSTraC curriculum for the acquisition of robotic-assisted surgery skills. RoSTraC provides a highly standardized, comprehensive and efficient training curriculum for RAS for surgical residents. We were able to prove in a prospective multicentric study the feasibility of the cloud-based learning approach for self-contained training of participants at their own institutional robotic system without permanent supervision. More importantly, we could demonstrate that participating surgical residents acquired fundamental and advanced RAS skills, and RoSTraC training led to significantly improved performance of advanced four-hand robotic procedures. Finally, we could confirm that all participants were successfully and safely embedded into the local surgical RAS team as shown by their advanced clinical practice during the last step of the curriculum.

## Data Availability

The data that support the findings of this study are available from the corresponding author [TK].

## Abbreviations

RoSTraC: Robotic Surgery Training Curriculum
LTB: Lübeck Toolbox
MIS: Minimally invasive surgery
RAS: Robotic-assisted surgery
VR: Virtual reality
